# Total hip arthroplasty via the direct anterior approach using a conventional traction table and fluoroscopy: a safe and cost-effective technique

**DOI:** 10.1051/sicotj/2024045

**Published:** 2024-11-19

**Authors:** Seiya Ishii, Tomonori Baba, Koju Hayashi, Yasuhiro Homma, Osamu Mutou, Muneaki Ishijima

**Affiliations:** 1 Department of Orthopaedic Surgery, Yokohama Tsurugamine Hospital Kanagawa Japan; 2 Department of Orthopaedics, Faculty of Medicine, Juntendo University Tokyo Japan; 3 Department of Medicine for Orthopaedics and Motor Organ, Juntendo University Graduate School of Medicine Tokyo Japan

**Keywords:** Direct anterior approach, Total hip arthroplasty, Fluoroscopy, Component positioning, Component safe zone

## Abstract

*Introduction*: Precise implant positioning during total hip arthroplasty (THA) is an important factor affecting dislocation rate and long-term implant survival. Although a special carbon fiber traction table for THA improves the accuracy of implant positioning, it is too expensive. We aimed to report the accuracy of cup positioning and complication rate in patients undergoing THA using a conventional noncarbon fiber traction table, which is generally used for osteosynthesis of femoral fractures. *Methods*: This retrospective study included 62 patients who received primary THA via the direct anterior approach using a conventional traction table with fluoroscopy between July 2022 and December 2023. Two observers recorded radiological outcomes using postoperative anteroposterior X-rays. The intraclass correlation coefficients of cup positioning angles were evaluated (inclination: 0.92, anteversion: 0.88 for intra-observer agreement; inclination: 0.91, anteversion: 0.84 for inter-observer agreement). Complications were defined as dislocation, periprosthetic fracture, ankle fracture, implant loosening, nerve injury, surgical site infection, deep vein thrombosis, and revision surgery for any reason. *Results*: Radiographic analysis showed an average cup inclination of 38.5° ± 4.3° (98.4% within Lewinnek’s safe zone). The average cup anteversion was 12.6° ± 4.6° (100% within Lewinnek’s safe zone). None of the patients experienced any complications. *Discussion*: A conventional traction table could be a feasible alternative to a carbon fiber traction table for performing THA via the direct anterior approach using fluoroscopy at general hospitals.

## Introduction

Total hip arthroplasty (THA) is an effective surgical technique for end-stage hip arthritis that provides excellent pain relief and improves quality of life [1]. The direct anterior approach (DAA) is a less invasive approach for THA. This approach can reach the joint via the innervated muscles without muscle transection, thereby accelerating recovery [2] and reducing the risk of dislocation [3]. The Judet brothers originally developed a traction table in 1985 [4], which facilitates femoral exposure owing to stable angulation and leg position that could be challenging to achieve in DAA-THA, particularly during the learning curve [5]. Subsequently, DAA-THA using a traction table has gained popularity owing to the pioneering traction table developed by Matta et al. which is specialized for DAA [6]. Carbon fiber-reinforced plastic is used in this traction table, which enables seamless intraoperative use of fluoroscopy. This enhances the accuracy of implant positioning [7, 8] and reduces the risk of intraoperative fracture [9]. However, the high cost of this carbon fiber traction table, typically around US$200,000 [10], limits its use in high-volume THA centers, thereby leaving other surgeons to perform DAA-THA without the traction table [11].

We hypothesized that a conventional non-carbon fiber traction table, which is commonly used in most centers for the open reduction and internal fixation of femoral fractures, could be a viable and cost-effective alternative to a specialized traction table for DAA-THA. However, the accuracy of cup positioning after THA using this conventional traction table has never been investigated. Thus, this study aimed to evaluate the accuracy of cup positioning in THA via the DAA using the conventional traction table compared to THA via the posterior approach (PA).

## Materials and methods

This single-center, retrospective cohort study was approved by the institutional review board of our hospital. We performed a power analysis using acetabular inclination and anteversion as the primary endpoints. Using a power of 0.9 and published data [12], we concluded that a sample size of at least 31 is needed in each cohort to detect a difference in anteversion of 5° or inclination of 5°. Therefore, we planned to include approximately 40 patients to allow for a generous dropout rate to ensure enough power for detecting primary endpoint differences. The secondary endpoint was acetabular safe zone distribution.

**Figure 1:**
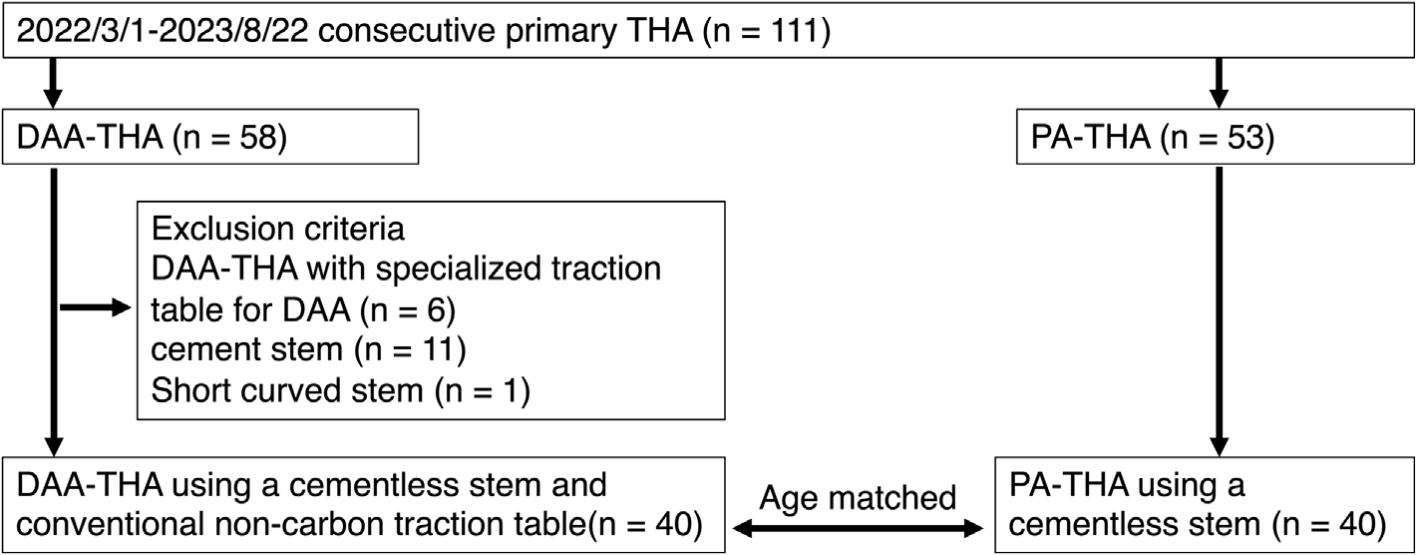
Study flow chart.

Figure 1 shows the study flow. This study initially included 111 consecutive patients who underwent primary THA via the DAA and PA between March 2022 and August 2023. In the initial phase of this cohort, DAA-THA was performed in six patients using a carbon fiber traction table specifically designed for DAA. However, due to the high costs associated with renting this specialized table for each use, its use was discontinued. Then, a conventional noncarbon fiber traction table was used to perform DAA-THA. This traction table was already equipped with our hospital and specifically used for osteosynthesis in case of femoral fractures. The exclusion criteria included patients who underwent THA using the carbon fiber traction table specialized for DAA-THA (6) and cases with cement stem (11), cementless short curved stem (1), After application of the exclusion criteria, 40 DAA-THAs using a cementless Avenir stem and an ordinary noncarbon traction table were included in this analysis, and 40 PA-THAs were analyzed as age-matched controls (Figure 1).

**Figure 2:**
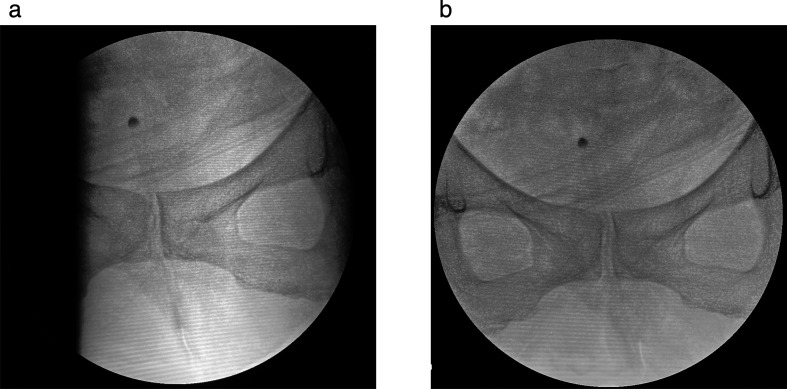
A technique to make bilateral obturator foramina visible on the C-arm monitor; (a) Metal structure under the pelvis obstruct fluoroscopic view; (b) Axial rotation of the pelvis and rotation of the C-arm make the bilateral obturator foramina visible.

All the DAA-THAs were performed by a single orthopedic surgeon (S.I). Patients undergoing DAA-THA were placed in the supine position on a standard surgical table, and both legs were secured with a conventional traction table used for osteosynthesis of femoral fractures (DR-6500 Takara Belmont Corp., Osaka, Japan). This instrument was manufactured from metallic materials, devoid of carbon components. A metal framework was placed beneath the pelvis, thus occasionally impeding the comprehensive visualization of the hip via fluoroscopy. In such cases, a small radiolucent pillow was placed under the hip. Axial rotation of the pelvis and fluoroscopy induced optimal pubic visualization without the interference of metal structures (Figure 2).

**Figure 3:**
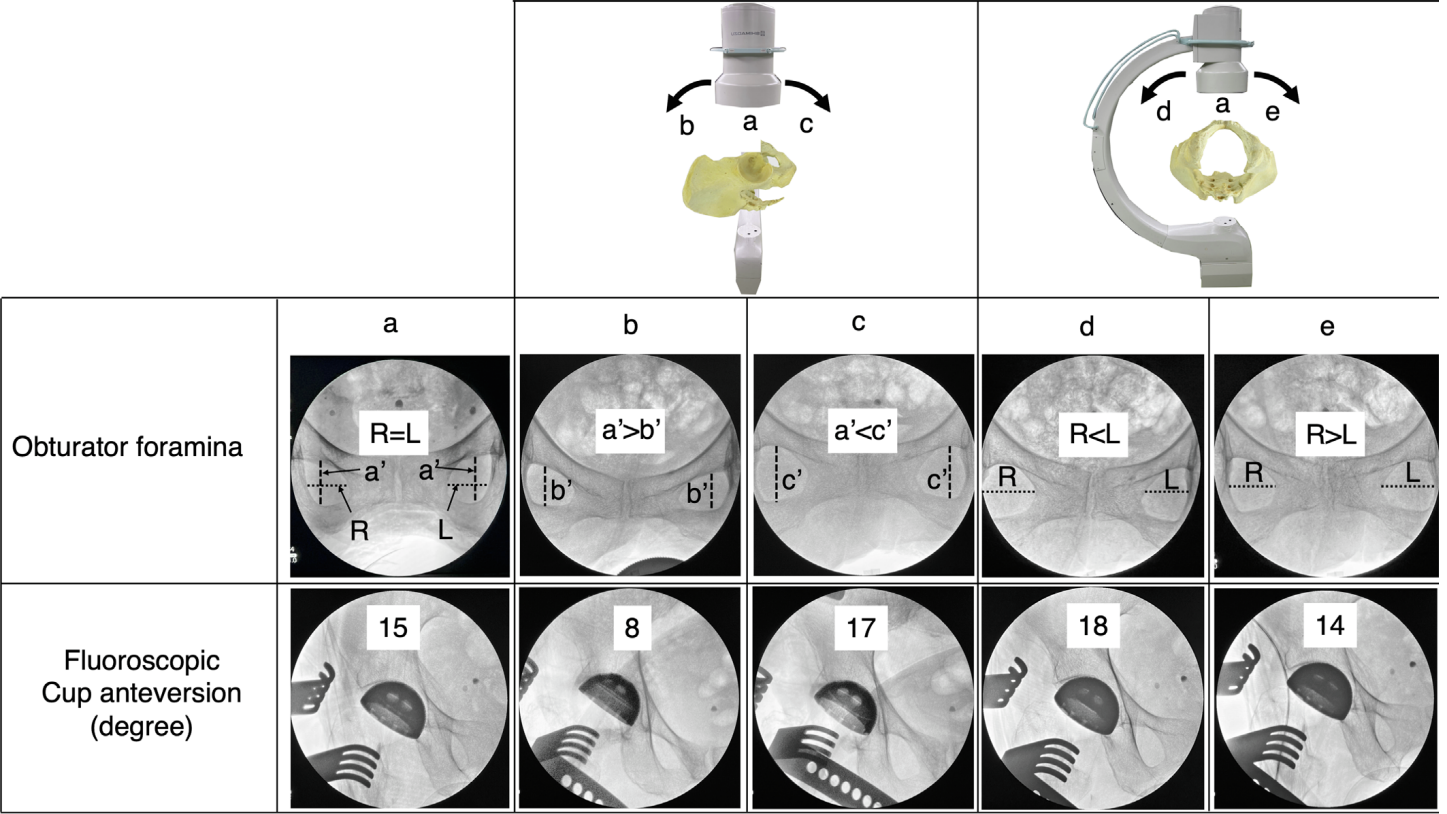
Changes in cup anteversion on the C-arm monitor depends on the angle of incidence of the radiation; (a) Optimal angle of incidence of fluoroscopy; (b), (c) Sagittal malrotation of the C-arm; a′, b′, c′, Each vertical length of the obturator foramina on the monitor a, b, and c; (d), (e) Axial malrotation of the C-arm; R, L, Each width of the obturator foramina on the right and the left side.

**Figure 4:**
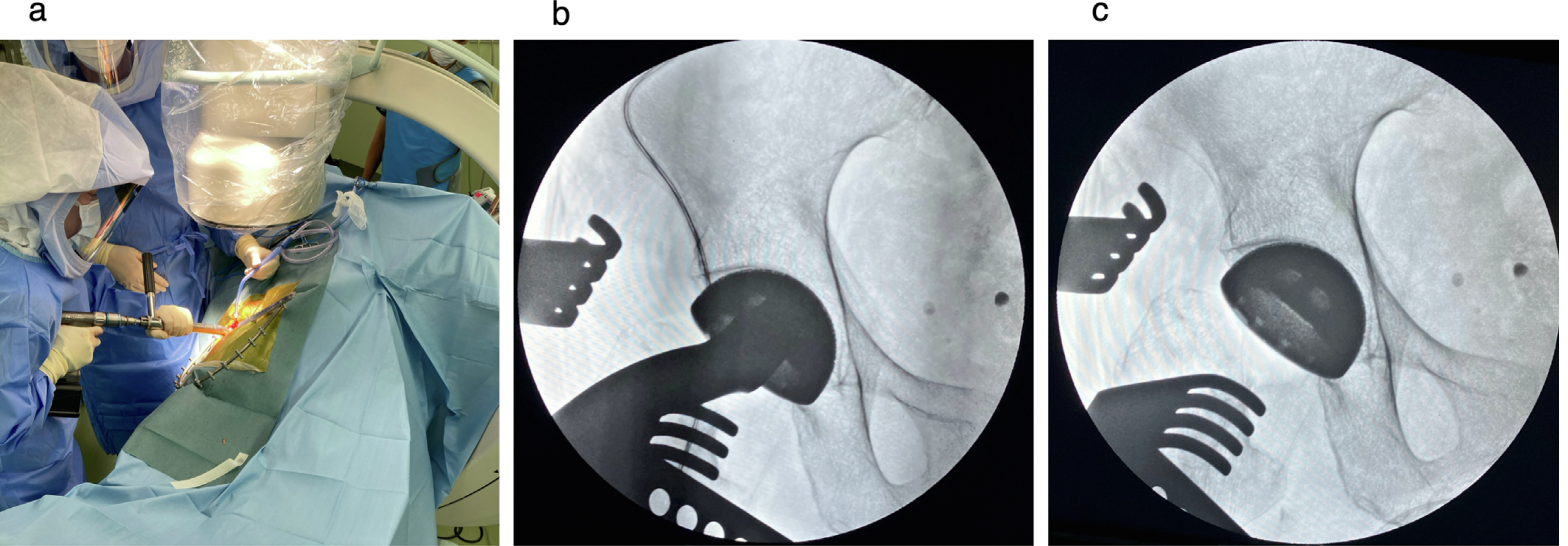
Acetabular procedure under fluoroscopic assistance; (a) Acetabular reaming; (b) Cup impaction; (c) Cup positioning within the optimal angle.

DAA-THA was performed as previously reported using the distal part of the Smith–Petersen approach [13]. Acetabular reaming, femoral osteotomy, stem, and cup placement were performed under fluoroscopic guidance. Before reaming and cup placement, the fluoroscopic angle perpendicular to the pelvis in the axial plane was evaluated by assessing the widths of both obturator foramina. Then, sagittal rotation was assessed by comparing the length of the obturator foramina along the body axis to the preoperative anteroposterior radiographs (Figure 3). Adjustment of the fluoroscopic inclination enhances the reproducibility of the implant positioning angle between intraoperative fluoroscopic images and postoperative radiograph [7]. The acetabular reamer was medialized into the acetabulum until adequate initial fixation was achieved while not penetrating the medial acetabular wall. After reaming the acetabulum, the cup was inserted at the optimal angle (Figure 4). For stem insertion, the traction table was extended so that the hip joint could be extended to 30°. Uncemented cups (G7 acetabular components, Zimmer-Biomet, Warsaw, IN, USA) and uncemented stems (Avenir complete hip system, Zimmer-Biomet, Warsaw, IN, USA) were used in all patients. A dual mobility (DM) system with the E1 Active Articulation bearing (Zimmer-Biomet, Warsaw, IN, USA) was selectively used in patients aged over 65 years.

PA-THA was performed based on the approach described by Moore by a single experienced orthopedic surgeon [14]. Patients underwent PA-THA in the lateral decubitus position. The acetabular cup was impacted into the acetabulum using an alignment guide. After fixing the optimal broach to the femur, a trial reduction was performed using a trial neck and trial head. After assessing hip stability, a single X-ray was taken to determine the implant positioning, neck length, and leg length. Finally, the stem and the head were inserted. Uncemented cups (29 cases; Tritanium, 11 cases; Trident 2, Stryker Orthopaedics, Mahwah, NJ, USA), uncemented stems (Accolade II, Stryker Orthopaedics, Mahwah, NJ, USA), and highly cross-linked polyethylene liners (X3, Stryker Orthopaedics, Mahwah, NJ, USA) were used in all patients. DM was not used in the PA group. For both approaches, all patients were allowed full weight-bearing immediately after the surgery except in cases where intraoperative fractures were evident.

As a clinical evaluation, we recorded operative time, intraoperative blood loss, complications (intraoperative femoral and acetabular fractures, infections, hip dislocations, and implant loosening), and revision surgery for any reason.

### Radiographic evaluation

Radiographic evaluation was performed using an anteroposterior radiograph in the supine position with both legs internally rotated by 10°. Postoperative radiographs were used to assess leg length, stem alignment, radiographic inclination, and radiographic anteversion of the acetabular component. Acetabular anteversion was calculated using a method described by Park et al. [15]. The acetabular component position was evaluated using Lewinnek’s safe zone (inclination target of 40° ± 10° and anteversion target of 15° ± 10°) [16]. The angle of the femoral component was measured as the angle between the femur and stem axis. All measurements were conducted using a computerized picture archiving and communication system (SYNAPSE; Fujifilm, Tokyo, Japan). The measurements were performed by two authors (S.I. and K.H.). The intraclass correlation coefficient (ICC) was used to measure inter- and intra-observer reliability. The measurements had a “good” to “excellent” ICC for intra-observer (Inclination; 0.95, Anteversion; 0.89) and inter-observer (Inclination; 0.95, Anteversion; 0.80) correlation [17].

### Statistical analysis

Continuous data except blood loss were presented as the mean and standard deviation along with the range, and categorical variables were expressed as the absolute and relative frequency. Normality was assessed using the Shapiro–Wilk test. An independent-sample t-test was used to compare continuous variables, except for blood loss. Because of non-normal distribution, blood loss was compared using the Mann–Whitney U test and presented as the median and interquartile range (IQR). Chi-square and Fisher’s exact tests were used for categorical variables. Statistical analyses were conducted using JMP Pro 17 for Macintosh. The tests were two-sided, and the level of significance was set at 0.05.

## Results

Forty patients each in the DAA and PA groups were finally included in the study analysis. The patients’ demographic data are presented in Table 1. Except for the etiology, no significant differences were observed in the basic characteristics of the patients between the two groups.

**Table 1:** Basic characteristics.

	DAA (*N* = 40)	PA (*N* = 40)	*P* value
Age (year)	71.1 ± 11.0 (51–94)	71.0 ± 9.5 (48–90)	0.944^†^
Height (cm)	155.3 ± 7.9 (135–172)	154.7 ± 9.0 (134–177)	0.723^†^
Weight (kg)	60.9 ± 14.4 (36–94)	57.0 ± 11.6 (39–86)	0.177^†^
Body mass index (kg/m)	25.1 ± 5.0 (14.4–37.2)	23.7 ± 3.9 (16.9–33.6)	0.165^†^
Gender, *n* (%)			0.165^†^
Male	6 (15.0)	5 (12.5)	1.000^§^
Female	34 (85.0)	35 (87.5)	
Etiology, *n* (%)			0.005^§^
Osteoarthritis	32 (80)	40 (100)	
fracture	8 (20) 0 (	0 (0)	
Operative side, *n* (%)			0.263^#^
Right	18 (45.0)	23 (57.5)	
Left	22 (55.0)	17 (42.5)	

* The values are given as the mean and the standard deviation (with the range in parentheses), or the number of patients (with the percentage in parentheses).

† Independent-samples *t*-test.

# Chi-square test.

§ Fisher exact test.

The clinical and radiographic outcomes are presented in Table 2.

**Table 2:** Clinical and radiographic outcomes.

	DAA N = 40	PA N = 40	*P*-value
Clinical outcome			
Operative time (min)	69.3 ± 15.0 (51–110)	91.7 ± 25.3 (61–170)	<0.001^†^
Intraoperative blood loss (mL)	137 (92–224)	253 (171–377)	<0.001^‡^
Total complication, *n* (%)	0	3 (7.5)	0.241^§^
Intraoperative fracture, *n* (%)	0	2 (5)	0.494^§^
Femoral fracture, *n* (%)	0	1 (2.5)	
Acetabular fracture, *n* (%)	0	1 (2.5)	
Infection, *n* (%)	0	1 (2.5)	
Dislocation, *n* (%)	0	0	
Implant loosening, *n* (%)	0	0	
Revision surgery, *n* (%)	0	2 (5)	0.494^§^
Radiographic outcome			
Cup alignment			
Inclination (degrees)	38.7 ± 4.4 (31–51)	36.6 ± 5.2 (25–47)	0.055^†^
Anteversion (degrees)	13.8 ± 4.8 (6–24)	16.1 ± 5.1 (8–29)	0.042^†^
Inclination within the “safe zone”, *n* (%)	39 (97.5)	35 (87.5)	0.201^§^
Anteversion within the “safe zone”, *n* (%)	40 (100)	37 (92.5)	0.241^§^
Both within safe zone, *n* (%)	39 (97.5)	32 (80.0)	0.029^§^
Stem alignment			
Coronal plane (degrees)	0.6 ± 1.6 (−2 to 5)	0.2 ± 1.7 (−4 to 4)	0.266^†^
Sagittal plane (degrees)	1.1 ± 2.1 (−3 to 6)	−0.2 ± 2.0 (−5 to 4)	0.006^†^
Coronal plane in neutral (−3 ≤ angle ≤ 3), *n* (%)	38 (95.0)	38 (95.0)	1.000^§^
Sagittal plane in neutral (−3 ≤ angle ≤ 3) , *n* (%)	35 (87.5)	37 (92.5)	0.712^§^
Leg length discrepancy (mm)	2.9 ± 5.3 (−9 to 16)	1.7 ± 5.6 (−10 to 15)	0.325^†^

* The values are given as the median and the interquartile range, the mean and the standard deviation (with the range in parentheses), or the number of patients (with the percentage in parentheses).

† Independent-samples *t*-test.

‡ Mann-Whitney U test.

# Chi-square test.

**Figure 5:**
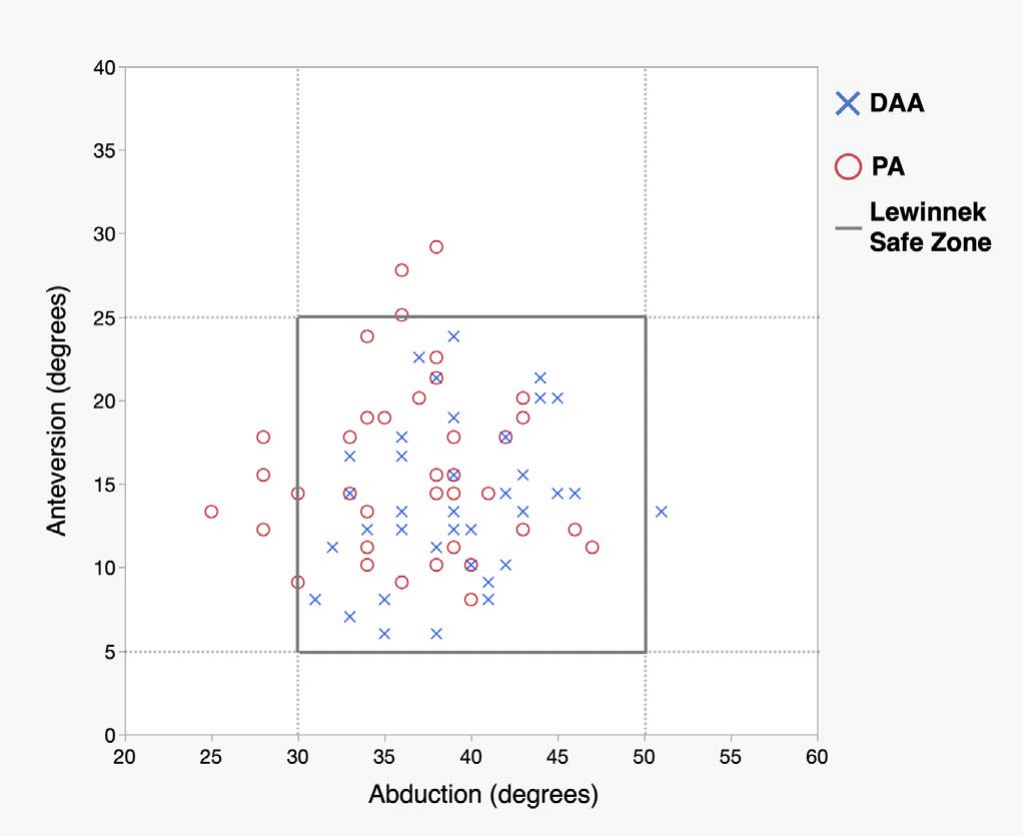
Distribution of cup positioning angle

As for the cup, the DAA group had a lower mean anteversion angle (DAA; 13.8° ± 4.8° vs PA; 16.1° ± 5.1°, *p* = 0.042) and a higher percentage of cup positioning angle within both of the safe zones (DAA; 97.5%, vs PA; 80.0, *p* = 0.029). (Figure 5). The PA group comprised one case each of intraoperative femoral and acetabular fractures.

There was no significant difference in leg length discrepancy (LLD) between the two groups. Regarding stem alignment, there were no significant differences between the two groups in the percentage of malalignment in either the coronal or sagittal planes. However, the mean sagittal angle of the stem was more flexed in the DAA group than in the PA group.

## Discussion

Accurate acetabular component positioning was performed in DAA-THA using a conventional traction table and fluoroscopy. This technique, which we term “Safe Anterior Approach with Fracture Table, SAAF”, can be performed without using expensive carbon fiber traction tables in various hospitals.

A previous systematic review showed that the intraoperative use of fluoroscopy for THA does not necessarily improve the accuracy of the cup positioning [18]; however, our study demonstrated a high incidence of cup placement within the safe zone. This discrepancy in the findings may be attributed to the following two factors: stabilization of the pelvis by the traction table and modification of the angle on fluoroscopy relative to the pelvis.

First, the traction table can reduce intraoperative pelvic motion and measurement errors using fluoroscopy. Pelvic motion during THA is difficult to avoid in any of the approaches, and intraoperative pelvic motion leads to an inaccurate assessment of the cup placement angle [19]. Shah et al. monitored the intraoperative pelvic movement in the supine position without a traction table, revealing that 91% of these patients experienced pelvic tilt in the axial plane. Moreover, when intraoperative pelvic axial tilt was not accounted for, a discrepancy in the estimated cup inclination exceeding 5° was noted in 32% of cases after the cup placement [20]. These data suggest that inaccurate cup positioning in previous studies could be induced by assessing cup positioning on the unstable pelvis. The traction table would minimize pelvic motion, reducing measurement errors with fluoroscopy.

Second, intraoperative pelvic rotation changes the incidence of fluoroscopic radiation against the pelvis and can alter the interpretation of the intraoperative cup placement angle [19]. Kobayashi et al. reported that DAA-THA induces a high cup anteversion angle compared to PA, due to sagittal rotation of the pelvis during the surgery [21]. Even with a fixed lower extremity, intraoperative traction causes the pelvis to slightly rotate in the sagittal and axial planes. Therefore, the fluoroscopic angle must be adjusted just before cup insertion.

The use of a conventional traction table in DAA-THA can have a cost-benefit, not only by eliminating the need for an expensive carbon fiber traction table but also by reducing the number of surgeons required to maintain the position of the lower extremity during surgery. DAA-THA with a traction table requires two surgeons, whereas PA-THA or DAA-THA without a traction table commonly requires three surgeons due to the need to hold the lower extremity. In the context of an aging population, orthopedic surgery is under increasing pressure to consider the economic impact of medical interventions. Expenditures for orthopedic surgeons represent a significant portion of healthcare costs, and the value of interventions within the healthcare system is being re-evaluated [22]. Therefore, the use of a low-cost conventional traction table, which allows DAA-THA to be performed by fewer physicians, may become an increasingly attractive and necessary option. The current study had several limitations. First, this was a single-center retrospective study, which limits the generalizability of the findings due to potential selection bias. A prospective, randomized controlled multicenter trial would provide a more objective evaluation of the effectiveness of our technique. Second, the control group for the DAA-THA using a conventional traction table was PA-THA. DAA-THA using a carbon traction table would be an ideal control group to demonstrate the non-inferiority of the DAA-THA using a conventional traction table. Third, we did not observe an increased risk of fractures or LLD in DAA-THA using a traction table in contrast to previous studies which have reported an association between the use of a traction table in THA and risk of intraoperative femoral fractures or LLD [11, 23]. We believe this is due to the consistent use of intraoperative fluoroscopy. If the femoral osteotomy level is too long, the risk of femoral fracture will be increased because the nature of neck anteversion induces the stem insertion in a flexed position [24]. To address this, we routinely use fluoroscopy to assess the neck length before and after femoral osteotomy. By accurately reproducing the preoperatively planned neck length under fluoroscopic guidance, the risk of intraoperative fractures and LLD will be decreased. The use of fluoroscopy during THA is an effective technique not only for accurate cup placement but also to reduce the risk of femoral fracture and LLD.

## Conclusion

The use of a conventional traction table with fluoroscopic guidance in DAA-THA is a cost-effective approach. Moreover, this approach has demonstrated a high accuracy in implant positioning. This innovation can facilitate the initiation of DAA-THA and improve safety across various institutions. Further large studies comparing carbon fiber and conventional traction tables should be performed to validate these results.

## Data Availability

Available upon request from the corresponding author.
